# A claim for plant health as a key component of the one health concept

**DOI:** 10.1016/j.onehlt.2025.101304

**Published:** 2025-12-18

**Authors:** Ramon Albajes, María M. López, Rafael M. Jiménez Díaz

**Affiliations:** a Agrotecnio Centre and Universitat de Lleida, Lleida, Spain; b Instituto Valenciano de Investigaciones Agrarias (IVIA), Centro de Protección Vegetal y Biotecnología, Moncada, Valencia, Spain; c Universidad de Córdoba, ETS Ingeniería Agronómica y de Montes, Córdoba, Spain

**Keywords:** Plant health, One health, Plant pest, Plant disease, Food security, Food safety

## Abstract

The concept of health has historically been more closely linked to the well-being of humans than to that of animals, plants or the environment. In contrast, the One Health concept, which emerged in recent decades, generally recognizes the interdependence of only three of its four components: humans, wild and domestic animals, and the environment, but plants have often been overlooked in this analysis. Because plant health has been undervalued within the One Health framework, we emphasize in this review its importance in ensuring food security and safety, two key issues in human and animal health, as highlighted in several of the United Nations SDG. Food production, marketing and consumption use a significant portion of the environment's natural resources, and plant health technology must ensure their sustainable use while safeguarding environmental health. We summarize the relationships between the four components of One Health, highlighting the development of antimicrobial resistance in human, animal and plant pathogens, and the resistance to plant protection products among plant pests, diseases and weeds. Three external drivers significantly influence plant health and One Health in the last decades: climate change, invasive alien species, and the international armed conflicts. The main reported effects of climate change on plant health include the shifts in distribution area, biology and life cycles of harmful organisms as well as plant-pest interactions. Another major factor compromising the sustainability of current plant health technology is the introduction and establishment of alien organisms affecting vegetables resulting from the increasing globalization of food trade, human labour and tourism.These challenges underscore the need to approach One Health at broader scales, beyond individual organisms or populations, as well as intensifying studies on plant health, to effectively address holistically the complex, interconnected risks affecting global health.

## Highlights

• The concept of health has historically been linked to humans.
• Plant Health has been undervalued within the One Health framework.
• Plants have a decisive influence on human, animal and environment health.
• Plant Health contributes to food security and safety, two key issues in One Health.
• Antimicrobial resistance in pathogens is one of the major public health threats.

## Graphical abstract

**Figure:**
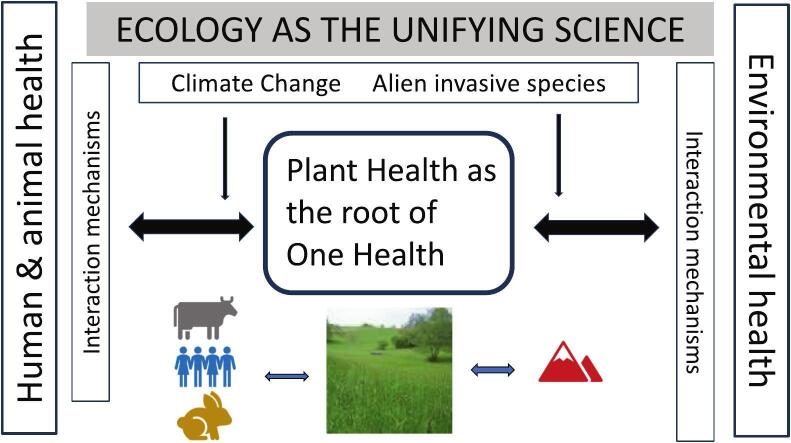

## 1 Introduction: a unified vision of health in the One Health perspective

### 1.1 The concept of health

The concept of health has historically focused on human well-being as a balance between people and the environment. The idea that disease has a natural aetiology was central to the understanding of health in ancient Chinese, Greek and Indian medicine, among others [1]. In the last century, the concept has expanded to refer to animals, plants and the environment in addition to humans, but the third United Nations (UN) Sustainable Development Goal (SDG), entitled Good Health and Well-Being, still deals solely with human health [2].

The concept of health differs widely when applied to humans, wild and domestic animals, cultivated and forest plants and the environment. The World Health Organization (WHO) defines health as “a state of complete physical, mental and social well-being and not merely the absence of disease or infirmity” [3] and also refers to the ability “to lead a socially and economically productive life” [3]. This definition essentially refers to the health of humans as individuals and members of populations [4]. However, a wide consensus on this concept is lacking [5].The World Organization for Animal Health (WOAH) [6] is concerned with the health and welfare of domestic and wild animals but does not define the concept of health in them, despite its reference to One Health. Most concepts of health in the plant health literature originate from research and practices aimed at protecting crops and forests from pests, which – according to the EU terminology – include diseases, phytophagous arthropods and weeds. A plant is regarded as healthy if its physiological performance, determined by its genetic potential and environmental conditions, is maintained [7]. Defining health in relation to the environment is more challenging: the European Environment Agency frames it primarily in terms of environmental quality and its impact on human health [8]. Nevertheless, the need for a holistic view of health problems has led the European Union to establish a commission through which several European agencies, including the European Environmental Agency, have developed an action plan to support the implementation of the One Health approach [9].

In this review, we aim to highlight that plant health is a vital component of the unifying One Health approach, and that its frequent exclusion from definitions and initiatives will undermine the overarching goal of this perspective, in the absence of a specific World Plant Health Organization.

## 2 Towards a unified concept of health: the One Health perspective

Although the health of people, animals, plants and environment is often addressed separately, there is growing awareness that they are linked and interdependent. Recognizing this interdependence requires a commitment to avoiding the prioritization of one aspect of health at the expense of the others. As stated by the WHO, in collaboration with the Food and Agriculture Organization of the United Nations (FAO), WOAH and the UN Environment Programme (UNEP), One Health is an integrated, unifying approach that aims to sustainably balance and optimize the health of people, animals and ecosystems. However, in this article, like many other authors, we distinguish plant health from environmental health, choosing not to group both under the broader term “ecosystems” [10,11]. The One Health perspective has two key advantages in addressing the complexity of multiple health challenges: first, it encourages the evaluation of costs and benefits across multiple domains; and second, it fosters innovation in service delivery by bringing together experts from different fields. There is now a broad consensus on the need to adopt a systemic human–animal–plant–environment approach to addressing today's health global challenges [12,13].

WOAH [14] identifies key facts that justify the need for unification of One Health, including the following: (i) 60 % of human pathogens originate from domestic or wild animals; (ii) 75 % of emerging infectious human diseases originate in animals; (iii) 80 % of pathogens identified as bioterrorism threats originate in animals; (iv) the FAO recently stated that global consumption of contaminated food causes more than 200 diseases; (v) zoonoses and contamination of plant products with human pathogens, mycotoxins and toxigenic fungi have an impact on human health; and (vi) antibiotic resistance is sometimes generated in chemically treated animals and plants.

In defining the One Health concept, it is important to emphasize that it does not simply represent the sum of four health domains. Rather, it is a unifying concept of health that also encompasses the disciplines necessary for its study and implementation. The broad basis of One Health requires a multidisciplinary and global approach to advance understanding and to implement ad hoc actions that bridge the gap between science and policy, enabling its full implementation to reduce health risks and impacts on humans, animals, plants and ecosystems [15]. Under this vision, the One Health Joint Plan of Action 2022–2026 [16] outlines the commitment of the FAO, UNEP, the WHO and WOAH to collectively advocate and support the implementation of One Health. The plan is especially valuable for integrating poor regions and countries in One Health networks, which are currently situated and resourced primarily in high-income countries. Moreover, it seeks to position One Health as a call for ecological equity, not merely health equity [17]. A similar initiative was undertaken by the European Union in late 2023: five health and environment agencies established a cross-agency One Health task force to contribute to the implementation of the One Health agenda [18].

## 3 One Health and the United Nations sustainable development goals

The implementation of the One Health approach is critical for achieving the UN 2030 Agenda for Sustainable Development and the related SDGs [19]. The human health component of the One Health approach is reflected in SDG 3 (Good Health and Well-Being), SDG 1 (No Poverty), and others. The health of plants, animals and the environment is reflected in SDG 6 (Clean Water and Sanitation), SDG 13 (Climate Action), SDG 14 (Life Below Water) and SDG 15 (Life on Land). Furthermore, the health of all living systems relies on the achievement of the SDGs tied to adequate services and resources, including SDG 2 (Zero Hunger) and SDG 11 (Sustainable Cities and Communities). Lastly, the effective implementation of the One Health approach involves the achievement of SDG 17 (Partnerships for the Goals).

In 2024, a study was carried out to measure the global and national degrees of implementation of One Health principles by scoring the value of more than 200 indicators in 160 countries/territories [20]. The rankings of indicators and sub-indicators were also compared to clarify the strengths and weaknesses of each region within One Health domains. The results showed that there is still much room for global improvement towards One Health approach at international level.

## 4 Improving food security and safety through plant health technology is a key component of the One Health perspective

To further explore the interrelationship among the four health components of One Health, we now consider how plant health influences the health of animals, humans and the environment.

### 4.1 Plants influence environmental health

Plants are a part of the environment and contribute to its health to the extent that they are healthy. Conversely, environmental health has a positive impact on the agroecosystems services that support plant health. The multiple interdependent mechanisms through which these mutual influences operate are summarized in Table 1. The biotic components of the environment and their functions are maintained thanks to the efficiency of the food web, which plants initiate by producing biomass through photosynthesis. Abiotic components also make the food web – and therefore life on the planet – viable. Much of the interrelationship between environmental and plant health occurs through plant health technology in agriculture or silviculture, as explained below.

**Table 1:** Relationships between environmental health and plant health in the One Health perspective. Mechanisms to reduce negative effects of plant health technology.

Environmental component	Related target in plant health	Mechanism or main actions to be taken	Source
Natural and seminatural habitats	Limit land use and deforestation	Increased crop productivity Diagnosis of landscape syndromes	[[21], [22], [23]]
	Promote habitat and food for wild animals, particularly pollinators and pest natural enemies	Nature conservation policy. Holistic consideration of agroecosystems and landscapes	
Atmosphere	Reduce the contribution of the global food system to greenhouse gas emissions	Control measures that reduce emission of greenhouse gases	[24]
	Mitigate impacts of climate change	Carbon absorption	[25]
	Increase oxygen provision	Increased photosynthesis in healthy plants	[26]
Soil	Enhance soil fauna and microflora	Organic soil management Organic crops	[23]
	Enhance soil microbioma	Pest and disease suppression Organic crops	
	Reduce erosion	Soil stabilisation by plant root system	
Water	Enhance the water cycle	Better penetration of rain water into soil	[27]
	Reduce consumption of fresh water by the global food system	More efficient irrigation systems compatible with maintaining plant health	[28]
Aquatic systems	Reduce eutrophication caused by the global food system	Optimized fertilization to avoid nitrogen and phosphorus pollution. Organic crops	[29,30]
Biodiversity	Enhance the food web	Increased quantity and quality of the first node species in the food web	[31]
Plants in urban areas	Enhance wellbeing of people living in urban areas	Planting of trees and shrubs in urban areas	[32]
Biotic and abiotic components	Reduce pesticide residue	Integrated pest management Organic crops	[33]
Other natural resources	Increase sustainability of plant health technology in crops and forestry	Increased efficiency in the use of natural resources	[21,34]
Full environment	Reduce food waste	Effective collaboration through a public-private partnership: e.g. donations, social supermarkets and food banks	[35]

### 4.2 Plants influence human and livestock health

#### 4.2.1 Plants feed the world: agricultural productivity and food security

Plants feed the world [36], and food security – the state of having reliable access to sufficient, safe, affordable and nutritious food at all times – is also a key aspect of One Health, because it is directly involved in human health: as stated above, it is a pillar of the UN SDGs. Table 2 summarizes the multiple aspects involved in the relationships between plant health and human and livestock health.

**Table 2:** Some examples of microorganisms that are pathogenic to plants, humans or domestic animals and which can be carried out, disseminated or multiplied by plants.

Pathogenic microorganism	Role of plants (host/carrier/ refuge/multiplier)	Effects on humans/ animals	Effects on plants or food	Source
*Claviceps purpurea*, *Aspergillus*, *Fusarium* and *Penicillium* spp. (fungi)	Carriers of mycotoxins	Toxic and allergenic mycotoxins	Pathogenic or non-pathogenic	[33]
*Epichloe* spp. (fungus)		Toxic mycotoxins	Endophyte	
*Burkholderia* spp. and *Pseudomonas aeruginosa* (bacteria)	Host	Disease in immune-compromised people or animals	Pathogenic	[37]
*Pectobacterium carotovorum* (bacterium)	Target of antibiotic treatments	Resistance to carbapenem antibiotics	Pathogenic	[38]
*Escherichia coli*, *Salmonella* spp.	Refuge for survival and source for dissemination through environment and food value chain	Pathogenic	To be studied	[39]
*Salmonella* spp. (bacterium)	Host: secondary colonizers	To be studied	Damage to plant tissues	[40]
Some phytopathogenic bacteria	Host	Antimicrobial resistance to several antibiotics	Antimicrobial resistance	[41]

Plants provide over 80 % of the food consumed by humans and are the primary source of nutrition for livestock. Therefore, agricultural and aquacultural activities, along with forestry, are key components of One Health throughout the entire food chain. In 2022, the FAO [42] estimated that the food intake of approximately 10 % of the world's population was below the level necessary to maintain health. It also estimated that this percentage may continue to increase as the demand of the growing world population increases, despite the dramatic decline in the population growth rate over recent decades [43]. Therefore, there is a need to significantly increase in a sustainable way agricultural productivity to meet the challenge highlighted by the FAO and to meet SDG 2.

#### 4.2.2 Plants feed the world: sustainability of plant health technology

The concept of sustainable development implies limits – albeit not absolute ones – that are imposed by the current state of technology and social organization on environmental resources, as well as by the biosphere's capacity to absorb the effects of human activities. A substantial part of the increase in agricultural productivity is due to improved agricultural practices, the development of high-yielding crop varieties, more efficient methods for safeguarding plant health from pests and reductions in food waste. However, the improved crop production and protection technologies that have driven substantial increases in agricultural yields have also given rise to the emergence and re-emergence of pests in recent decades, raising concerns about the sustainability of the current plant health technology [33].

The sustainability of plant health technology is affected mainly by two of its own effects that also have an impact on environmental, human and animal health (Table 1, Table 2): the development of resistance to plant protection products in many pest species; and resistance to antimicrobial substances – particularly antibiotics – in bacterial pathogens. More than 500 species of arthropod pests are recorded as resistant to one or more active ingredients of plant protection products [44,45]. Approximately 700,000 human deaths per year worldwide are attributable to infections caused by drug-resistant pathogens [41]. The WHO has declared antimicrobial resistance to be one of the top ten global public health threats facing humanity, and this has been called a “quintessential” One Health issue [41].

#### 4.2.3 Plants feed the world: food safety in relation to plant health

In addition to contributing to the development of antimicrobial resistance, crop pests also harm food safety from a One Health perspective, as harvests can act as carriers of enteric human pathogens and harmful microbial-based toxins [33,47]. For example, foodborne illnesses represent a serious global burden on human health, reportedly affecting 600 million people and accounting for 33 million disability-adjusted life years in a single year [46]. Although human and animal pathogens are not pathogenic to plants, contaminated plants can serve as a niche for their survival, or even multiplication, in the absence of a host, or disseminate them through the environment and food supply chains. The complex interrelationships between plants and both human and animal health are well illustrated by the adverse effects of pest- and disease-affected crops on humans and livestock. Some of these cases, mainly involving bacteria and fungi, are listed in Table 2. Some other mechanisms through which plant health technology may affect food safety are also mentioned in Table 3.

**Table 3:** How plant health and its technology influence human and livestock health through food security and safety in the One Health perspective. PPP: plant protection products.

Stage in the agroforestry value chain	Objective	Current situation	Mechanism or action to be taken	Source
Whole value chain	To use products obtained from agriculture	Plants provide 80 % of food consumed by humans and livestock	Increased accessibility to data about production, storage, trade and consumption of food	{36]
	To reduce food waste UN SDG 12	Food waste in EU is between 70 and 100 kg/capita/year mostly in households	Effective collaboration through a public-private partnership: e.g., donations, social supermarkets and food banks	[35]
	To prevent contamination of agroforest harvest by human and livestock pathogens	Foodborne illnesses reportedly affect 600 million people	Use of sanitary measures throughout the value chain	[33,46,47]
	To improve One Health and global health	Study and intervention of human, animal, plant and environmental health are fragmentary	A unified scientific approach to plant health, facilitating its integration in a One Health framework	[48,49]
Food production	To produce the necessary food quantity and quality for everybody	10 % of world population are undernourished	Increased agricultural productivity and development of more nutritive food	[43]
	To reduce crop losses in pre- and postharvest	32 % of crop yield loss caused by pests. Current pest control measures are insufficient	More efficient pest control measures	[50,51]
	To prevent the development of resistance to plant protection products (PPPs)	Worldwide, at least 500 insect pest species have developed insecticide resistance. 273 weed spp. have been recorded as resistant to herbicides	Rotational use or mix of active ingredients Reduction of use of PPPs Organic farming	[52,53]
	To prevent the development and dissemination of resistance to antimicrobial products	Annually, 700, 000 deaths in the world are attributable to infections by drug-resistant pathogens	Elimination or reduction in use of antibiotics in agriculture and livestock. Prevention of circulation of resistance genes in the environment	[41,54]
	To avoid the exposure to negative abiotic conditions	Variable climate	Cultural practices Adapted cultivars	[55]
	To reduce the use of synthetic PPPs	Use of PPPs is stable in most of the world	Development of non-chemical control measures. Organic farming	[56]
	To reduce the use of synthetic PPPs	10.000 people die each year in developing countries from pesticide poisoning and about 400,000 suffer acutely	Development of Integrated Pest Management. Reduction of chemical residues in food. Reduction of PPP toxicity	[57,58]
Food consumption	To prevent contamination of crop harvest by human and livestock pathogens	Foodborne illnesses reportedly affect 600 million people	Use of sanitary measures throughout the value chain	[47]
	To optimize human and livestock nutrition	Unhealthy feeding	Improvement of healthy feeding education for humans using healthy plants and animal food	[19]

## 5 External drivers influencing plant health and One Health

### 5.1 Climate change

The main reported effects of climate change on plant health that also have consequences for One Health include (i) the distribution area of phytophagous arthropods, pathogens and weeds; (ii) the biology of phytophagous arthropods and the life cycle of pathogens; (iii) the trophic interactions regarding the population dynamics of phytophagous species and the physiology of plant–pathogen interactions; and (iv) the efficiency of the strategies used for disease control [33,60]. Climate change also affects international trade flows of plants and plant products and will change the infectivity, severity and distribution of plant pests [55]. One review [59] found that anthropogenic climate change has affected 82 % of the 94 core ecological processes identified, ranging from genetic diversity to ecosystem functioning.

Most research on the consequences of climate change for plant pests has been performed in the laboratory, in free-air CO_2_ enrichment facilities or in phytotrons. A more realistic approach to studying these consequences is to assess the prevalence, incidence and progression of plant pests along an elevation gradient from low to higher altitudes of the host's distribution range (with a constant photoperiod). Complementary research can be conducted in different habitats across a latitudinal gradient (with a variable photoperiod), accompanied by meteorological data collection at each site. Long-term big data are essential for determining the influence of factors related to climate change. Various types of models have also been used to predict the future impact of climate change on plant pests and thereby determine the efficacy of different tactics for mitigating the crop losses they cause [60]. However, there are still many gaps and uncertainties regarding the impacts of climate change on food production because of the large number of arthropod, phytopathogenic and weed species affecting plant health or production, which warrant further research efforts.

### 5.2 Invasive alien species

Alien species are exotic animals, plants and microorganisms that originate from other regions and can have a significant impact on ecosystems, as well as on agriculture, silviculture, livestock and other sectors. The introduction and establishment of alien pests is a main consequence of the increasing globalization of food trade, human labour and tourism, and is another factor compromising the sustainability of current plant health technology [33]. Climate change may also favour the introduction and establishment of alien pests and the expansion of their geographic range. Alien species are considered invasive if they are capable of displacing endemic or native species. They include species that, as a result of human activities, have moved into a region outside their native habitat and established a self-sustaining population, with negative impacts for local biodiversity and ecosystems.

The severe global threat – especially to biodiversity – posed by alien species introduced into new habitats is underappreciated, underestimated and often unacknowledged. According to a report by the Intergovernmental Platform on Biodiversity and Ecosystem Services [61], more than 37,000 alien species (including 3500 invasive ones that seriously threaten nature) have been introduced by human activities into new regions and biomes around the world. This impact of alien species affects all four components of One Health in various ways and through multiple interactions.

The increased impact on health arising from the introduction of alien and invasive pathogen vector species and changes in their distribution has been studied most extensively in the fields of human and veterinary medicine [62]. However, the introduction and establishment of phytophagous arthropods, pathogens and weeds has also significantly affected plant health. It is estimated that up to 15 % of more than 10,000 alien species present in the EU countryside, waterways and marine environments are potentially dangerous to European biodiversity [63].

### 5.3 International armed conflicts

International armed conflicts seriously impact all components of One Health by destroying infrastructures, interfering services, displacing people and creating conditions to increase exposure of populations to disease risks. The recent conflict in Ukraine has demonstrated how the disruption of agriculture and the agricultural industry in a single country has hampered the availability of products from cultivated plants such as several cereals or sunflower oil or fertilizers for various crops at global level, especially at the European Union. Indirect and long term incidence of other armed conflicts on One Health components still need to be investigated.

## 6 The scientific basis of plant health for a multidisciplinary approach to One Health

### 6.1 A complex scientific approach to the concept

Because One Health developed mainly from studies and practices of human medicine, it has been assumed that these disciplines also constitute the main scientific basis for One Health research. However, human health researchers have long realized that impairment in the health of both wild and farmed animals, degradation of food systems, environmental deterioration and loss of biodiversity all have an impact on human health and wellbeing. Therefore, the integrative One Health approach recognizes that health depends on the state of the entire organization of life; consequently, disregarding scales broader than individual organisms or even populations is a serious misconception in One Health research [22]. A perspective of this type integrating agricultural and ecological sciences has been advocated for plant pathology (and other disciplines concerned with plant health) in order to address emerging food security and environmental challenges [48]. Progress in the field of One Health requires a collaborative, multidisciplinary international approach that transcends the boundaries of animal, human, plant and environmental health [64]. Ecology should be the unifying science that integrates knowledge and understanding of the Earth, as well as the animal–human–plant–environment connections within it [65].

### 6.2 Is a scientific basis for plant health available for integration in One Health?

Whether plant health has a unifying scientific basis that could underpin research, training and actions for protecting plants from pests is a question that needs to be addressed. Such a basis would benefit the objectives, methods and practical applications of plant protection. Also, having a common scientific discipline for all plant health research would facilitate the integration of plant protection practices with agronomy, food quality science and ecology [48]. Unfortunately, a reductionist approach to plant health has led to an increasing specialization of various branches of biology, running counter to the need to integrate plant protection problems into One Health. Consequently, plant health science is characterized by a proliferation of subdisciplines that often focus on just one pest organism neatly classified within a taxonomic system, while overlooking interactions with other organisms or insights from other branches of biology [7,33]. The clear need for a holistic unifying vision of plant health is even more evident when researchers seek to develop integrated pest management programmes, whose application to crops must be combined with the other disciplines involved in the entire food value chain [66,67].

Several authors have proposed plant medicine (or phytiatry) as a discipline encompassing all research, training and technology transfer in the field of plant protection [[68], [69], [70]]. The application of the term medicine to plant health offers clear innovative value, but we must avoid simply transferring the principles and strategies of human medicine to plant medicine.

## 7 Conclusions

The role of plants in a One Health perspective has been discussed by several authors, although in different frameworks and with diverse objectives [12,13,71]. Hoffmann et al. [71] discuss the findings of a survey conducted among specialists aimed at understanding the implications that agricultural plant protection practices may have on the related One Health components, particularly within the context of the agriculture of a developing African country. Gullino et al. [12] highlight the importance of plants for ensuring global well-being, particularly during times of human health problems or economic crises, or during international armed conflicts. Also, these authors [12] together with Hoffmann et al. [71] show that these situations further emphasize the relationships between the four components of One Health and that their preservation is essential for guaranteeing global well-being. Lorenzini and Nali [13] also highlight the lesser role that plant health has historically played in the conceptual development of One Health. To counter this undervaluation of plants within the One Health framework, these authors review how various crop diseases affect the health of people, animals, and the environment. In the review we have incorporated several of the relationships between plants and other components of health mentioned by the authors cited in this paragraph. In addition, we have chosen to justify the importance of plants as the foundation of the One Health concept by starting with the conceptualized term “Health” and, from there, deriving the symbiotic relationships among the four components of One Health. The following conclusions justify why plant health must be considered at the root of One Health.

i. *The four components of One Health*. Human health has often been defined as a separate concept, but a broader definition of health must be fostered to include domestic and wild animals, plants and the environment. This is the goal of the One Health approach, which recognizes the interdependence of the four components, all stemming from a common root: plant health.
ii. *Plants and environmental health*. Plants are a part of the environment and contribute to its health to the extent that they are healthy. Conversely, environmental health has a positive impact on the agroecosystems services that are supported by plant health
iii. *Plants are central to food security and safety, and to ecosystems*. Agriculture and silviculture support health but also have an impact on humans, animals and the environment. Therefore, a shift towards a more sustainable technology in the agrifood systems is required.
iv. *Plant health technology and One Health*. Plant protection is progressing under the One Health perspective by increasing food safety, reducing the impact on environmental health and reducing food contamination with pathogens or their products that harm human and animal health.
v. *Major challenges of current plant health technology.* The development of resistance to plant protection products in pests and antimicrobial resistance in plant, human and animal pathogens must be curtailed.
vi. *Climate change* has an impact on plant health both directly by influencing pest biology and control and indirectly by affecting crop and forest ecology, susceptibility and yield.
vii. *Globalization* facilitates the transboundary spread of alien pests, thus threatening plant health and impairing One Health in some cases.
viii. *Armed conflicts* can severely impact all components of One Health by many different ways, depending on the countries involved.
ix. *One Health* is multidisciplinary and research on it must focus on ecological interactions among the four health components.
x. *Plant medicine* has repeatedly been proposed as a more comprehensive approach to the science of plant health, but its foundations are different from those of human and animal medicine if we do not start from the basic concept of Health.

## CRediT authorship contribution statement

The three authors contributed to writing and conceptualization.

## Declaration of competing interest

The authors declare that they have no known competing financial interests or personal relationships that could have appeared to influence the work reported in this paper.

## Data Availability

No data was used for the research described in the article.
